# Blockade of 5-HT2A receptors inhibits emotional hyperthermia in mice

**DOI:** 10.1007/s12576-019-00703-7

**Published:** 2019-08-20

**Authors:** Vanshika Sinh, Youichirou Ootsuka

**Affiliations:** Centre for Neuroscience, College of Medicine and Public Health, Flinders University, GPO Box 2100, Adelaide, SA 5001 Australia

**Keywords:** 5-HT2A, SR-46349B, Body temperature, Heart rate

## Abstract

This study determined whether blockade of 5-hydroxytryptamine 2A (5-HT2A) receptors attenuated hyperthermia and tachycardia responses to psychological stress in mice. For this purpose, male mice (C57BL/6N) were pre-instrumented with a telemetric probe to measure core body temperature and heart rate prior to experimentation. Vehicle or 5-HT2A antagonist, eplivanserin hemifumarate (SR-46349B) ((1Z,2E)-1-(2-fluorophenyl)-3-(4-hydroxyphenyl)-2-propen-1-one O-[2-(dimethylamino) ethyl] oxime hemifumarate) (0.5, 1.0, 5.0 mg/kg), was injected intraperitoneally. To elicit psychological stress, an intruder male mouse confined to a small cage was introduced into the resident mouse’s cage 30 min after administration of the injection. The application of this psychological stress increased body temperature by ~ 1.0 °C and heart rate by ~ 150 bpm in the vehicle group. In contrast, SR-46349B was shown to reduce this psychological stress-induced increase in body temperature in a dose-dependent manner (*P* < 0.05). However, the SR-46349B treatment groups had no influence on the intruder-elicited increase in heart rate. This study, therefore, suggests that 5-HT2A receptors play a significant role in mediating hyperthermia, but not tachycardia, during intruder-elicited psychological stress.

## Introduction

When animals are faced with stressful stimuli, they respond by changing aspects of their physiological function. These changes include an increase in body temperature, a response referred to as emotional hyperthermia [1, 2], as well as an increase in heart rate [3]. A body of evidence supports the view that serotonin (5-hydroxytryptamine, 5-HT) is one of the neurotransmitters involved in this thermoregulatory control [4–7]. More specifically, previous studies have shown that serotonin 2A receptors (5-HT2A) are involved in thermoregulatory control through the central nervous system [8, 9]. Blockade of 5-HT2A receptors has been demonstrated to attenuate thermogenesis in brown adipose tissue elicited by restraint stress [10] and hyperthermia by social defeat stress [9] in rats. These studies argue that blockade of the 5-HT2A receptors does not affect tachycardia elicited by these stress models [9, 10], despite the serotonergic system having a defined role in cardiovascular control [11–14].

There are several models that exist to study stress. In the restraint model, an animal is confined to a small, cylindrical tube, severely restricting their physical movement. Alternatively, the social defeat stress paradigm involves physical conflict between the resident and intruder animal. Fundamental to both of these stress models is the inclusion of physical stimulation, which may induce a qualitative change in the stress, making it difficult to account purely for psychological stress. It has, therefore, been difficult to ascertain the exact role of 5-HT2A receptors in the physical output of psychological stress. In the present study, we addressed this issue using an intruder–resident model, in which the resident animal is suddenly confronted with an intruder animal confined in a small transparent cage. Since there is no physical contact between the animals, this model induces psychological stress in the absence of physical stimuli. Using this model, we determined whether the 5-HT2A receptor antagonist, eplivanserin hemifumarate ((1Z,2E)-1-(2-fluorophenyl)-3-(4-hydroxyphenyl)-2-propen-1-one O-[2-(dimethylamino) ethyl] oxime hemifumarate) could inhibit the physiological outputs of hyperthermia and tachycardia characteristic of psychological stress.

## Materials and methods

The study was conducted on male C57BL/6N mice (*n* = 7, body weight 25–40 g) bred at the Flinders University in accordance with the Australian Code for the Care and Use of Animals for Scientific Purposes (8th edition) and with ethical approval from the Animal Welfare Committee of Flinders University.

### Surgery

Mice were bred in a grouped manner. On the day of the surgery, the animals were transferred to a surgical room and anaesthetised using a general anaesthetic (2% isoflurane, Veterinary Companies of Australia, Sydney, Australia) combined with 0.8 L/min oxygen, along with analgesia (Caprofen 0.1 ml, 5 mg/kg, Norbrook Laboratories, Newry, UK) and antibiotics (Baytril 0.1 ml, 15 mg/kg, Bayer Aust, Sydney, Australia) being administered prior to surgery. A telemetry probe (ETA-F10, Data Sciences International (DSI), Saint Paul, USA) was surgically implanted in the intraperitoneal cavity of the mice to measure core body temperature and electrocardiogram (ECG) [15]. The electrical leads for ECG were passed subcutaneously so that they sat between the connective tissue and skin, with the negative electrical lead sitting at the left breast muscle and the positive at the right leg. After surgery, each mouse was housed individually in a cage and allowed to have a week’s recovery before experimentation commenced. The cages maintained a 12 h/12 h light–dark cycle (on at 0700 and off at 1900).

### Drug administration

The selective 5-HT2A receptor antagonist, eplivanserin hemifumarate ((1Z,2E)-1-(2-fluorophenyl)-3-(4-hydroxyphenyl)-2-propen-1-one O-[2-(dimethylamino) ethyl] oxime hemifumarate) (SR-46349B, Tocris Bioscience, Bristol, UK) was dissolved in dimethyl sulfoxide (DMSO) (Sigma-Aldrich, St. Louis, USA). The concentration of DMSO was 2.8% of the final volume after dilution with ringer to make up a final volume of 0.4 ml, making the amount of DMSO and ringer consistent across all drug doses (including vehicle).

### Experimental procedures

The mice were exposed to daily habituation to the restraint technique used in the injection process, beginning 5 days prior to experimentation.

One day prior to each experiment, the resident mouse was placed in a temperature-controlled recording chamber maintained at around 26 °C. Food and water were available ad libitum during experimental recording. On the experimental day, between 9 and 10 a.m., either SR-46349B or vehicle was administered intraperitoneally. Each mouse was subjected to three different doses of SR-46349B (0.5 mg/kg, 1 m/kg, 5 m/kg) and vehicle. At least, 3 days recovery period was given between each injection. To avoid serial effects, a rotational design was used. Thirty minutes after the administration, an intruder male mouse (the same strain) confined to a small, transparent cylindrical container was placed in the resident cage for 30 min. The intruder mouse was then removed. A new intruder mouse was used every time to avoid habituation effects.

### Data recording and analysis

Body temperature and ECG signals from the telemetry probe were detected with a PhysioTel Receiver (RPC-1, DSI) and a communication box (Matrix 2.0, DSI) and were in turn captured by LabChart (ADInstruments, NSW, Australia) at 1 Hz via an analogue–digital converter (Powerlab, ADInstruments). Locomotor activity was detected with a passive infrared sensor (NaPiOn, AMN1111, Panasonic, Osaka, Japan) [15] and digitized at 100 Hz. Activity was expressed as the total amount of movement detected (sec) per min. Data were then imported into IgorPro (WaveMetrics, OR, USA) for further analysis.

Statistical analysis was performed using SPSS (IBM, NY, USA). The magnitude of the intruder or drug-elicited response was measured as the area under the curve (AUC) from 1 to 25 min after intervention. Pre-administration values were measured as the average of 5 min prior to administration of the injection. Alternatively, pre-intruder values were measured as the average of 5 min prior to the introduction of the intruder. The difference between these pre-intervention values and post-intervention individual time points was assessed with a paired *t* test. Along with this, the linear regression between the log-dose and the AUC was calculated to assess a dose-related response for SR-46349B. If the log-dose response was not seen to be significant, repeated measures one-way ANOVA was performed with a Least Significant Differences (LSD) post hoc test to determine any significant differences between the four treatment groups, including vehicle. The significance level was set to *P* < 0.05.

## Results

**Fig. 1:**
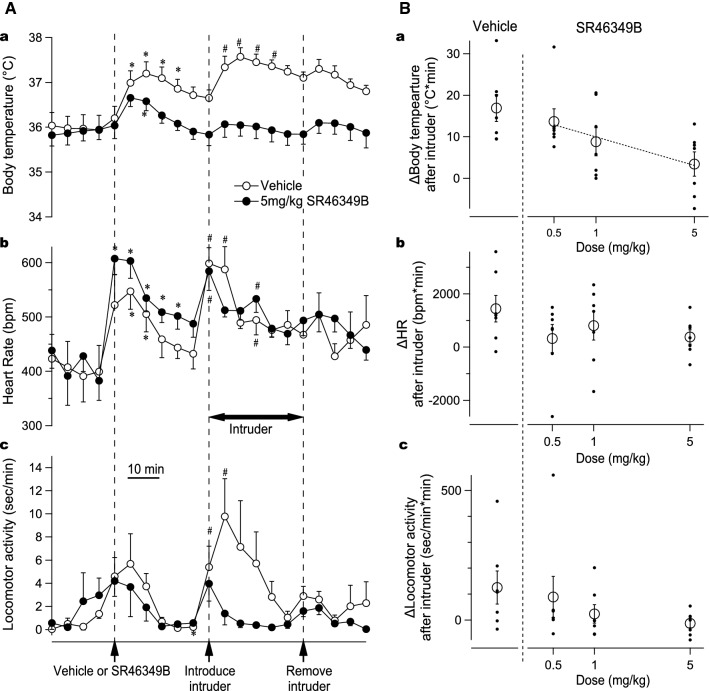
Effect of 5-HT2A antagonist on the intruder-elicited increase on body temperature and heart rate. **A** Averaged physiological recorded data from the resident mice after vehicle (2.8% DMSO, 0.4 ml) or SR-46349B (5 mg/kg i.p.) showing body temperature (a), heart rate (b) and locomotor activity (c). The administration was made 30 min before the introduction of the intruder mouse into the home cage of the resident mouse. The caged intruder was removed 30 min after introduction. Asterisks indicate a significant difference from baseline value before injection *P < 0.05. Hashtags indicate a significant difference from baseline value prior to the introduction of the intruder ^*#*^*P* < 0.05. **B** Log-dose regression analysis of drug effect on the intruder-elicited response on body temperature (a), heart rate (b) and locomotor activity (c). Each black dot represents area under the curve (AUC) during 30 min after the introduction of the intruder for individual cases. Each transparent circle represents the mean value of the cases for each treatment (*n* = 7, mean ± SEM). Dashed line in (a) indicates significant linear regression between the log-dose of SR-46349B and the intruder-elicited changes in body temperature *P* < 0.05

Figure 1A shows the group data after administration of vehicle (2.8% of DMSO in 0.4 ml Ringer, i.p.) or the highest dose of SR-46349B (5 m/kg in 2.8% of DMSO, 0.4 ml, i.p.) and the subsequent introduction of the caged intruder mouse.

### Effect of SR-46349B on baseline temperature, heart rate and locomotor activity

After vehicle or drug administration, body temperature increased by ~ 1.0 °C (Fig. 1A-a) and heart rate increased by ~ 170 bpm (Fig. 1A-b). Locomotor activity increased by ~ 6 s/min (Fig. 1A-c). These parameters then decreased back towards the pre-injection levels. In the vehicle control group, body temperature did not fully return to the pre-injection level (Fig. 1A-a). The AUC was measured to assess the influence of the injection administration on each parameter and compare between each treatment group. There was no significant effect of SR-46349B on the magnitude in all three dose cases (P > 0.05) (*F*(3,18) = 2.476 for body temperature; *F*(3,18) = 0.483 for heart rate; *F*(3,18) = 0.911 for locomotor activity).

### Effect of SR-46349B on intruder-elicited increase in body temperature, heart rate and locomotor activity

In the vehicle treatment group, the introduction of the intruder mouse elicited an increase in body temperature by ~ 0.9 °C and in heart rate by ~ 150 bpm (Fig. 1A). Locomotor activity increased by ~ 10 s/min (Fig. 1A). When response magnitude in each parameter was assessed by calculating AUC, SR-46349B attenuated the intruder-elicited increase in body temperature in a dose-dependent manner (log-dose regression, (*F*(1,19) = 5.44, *R*^2^ = 0.223, *P* < 0.05) (Fig. 1B-a). In contrast, SR-46349B did not cause a significant effect on the intruder-elicited increase in heart rate and locomotor activity (P > 0.05) (*F*(3,18) = 1.343 for heart rate; *F*(3,18) = 1.866 for locomotor activity) (Fig 1B-b, B-c).

## Discussion

Unique to this study is the use of the resident-intruder model. The use of physical and psychological stressors in conjunction, such as in restraint and social defeat interventions, exists as an inherent limitation to the study of psychological stress. By isolating the psychological stress response, this study provides an important perspective to further the understanding of emotionally elicited stress. The introduction of the intruder increased body temperature and heart rate in the resident mice. While pharmacological blockade of the 5-HT2A receptors substantially inhibited the hyperthermic response, it did not affect the tachycardia response. These findings mirror those found in similar studies using restraint and social defeat models and, in doing so, provide further evidence that 5-HT2A receptors are involved in emotionally triggered hyperthermia but not cardiac control [9, 10].

This study took important steps to avoid the impact of experimental limitations that could influence the significance of the results. Relevant to this is the use of dimethyl sulfoxide (DMSO) in the experiment. DMSO is widely used as an organic solvent for biological applications and, itself, has a hypothermic effect [16, 17]. A previous study shows that 2 ml/kg of undiluted DMSO does not cause a significant effect on body temperature in mice [16]. With this in mind, a concentration of 2.8% DMSO, which is equivalent to 0.4 ml/kg of undiluted DMSO, was used in our study so that it would not influence body temperature.

Blockade of 5-HT2A receptors did not cause significant changes in locomotor activity. This result suggests that 5-HT2A-induced inhibition of emotional hyperthermia is not simply due to a change in locomotor activity. Intruder-elicited hyperthermia is seen to be reversed by antipsychotic agents without major inhibitory effects on this behavioural activity [18]. Importantly, emotional hyperthermia can occur with freezing behaviour [19] or under stress that restricts behaviour [20].

While this study was not designed to establish the location of the 5-HT2A receptors responsible for driving these sympathetic thermoregulatory outputs, there is strong evidence to suggest that these actions are centrally mediated. There is expression of 5-HT2A receptors in the intermediolateral column of the spinal cord, where the preganglionic neurons reside [21]. Our previous studies have demonstrated that 5-HT2A receptors in the spinal cord, but not in the autonomic ganglia, mediate cutaneous vasoconstriction as a function of the sympathetic output of thermoregulation [22]. It is possible that 5-HT2A receptors involved in thermogenesis in brown adipose tissue are in a similar location since microinjections of 5-HT into the intermediolateral column cause thermogenesis [23].

The dorsomedial hypothalamus is known to play a crucial role in emotional hyperthermia [24]. Some anatomical studies have reported expression of 5-HT2A receptors in the hypothalamus of rats [21, 25, 26]; however, it remains to be investigated whether activation of 5-HT2A receptors in the dorsomedial hypothalamus is involved in emotional hyperthermia.

Serotonergic neurons are abundant in the medullary raphé nuclei which project to the intermediolateral cell column [27]. Within this, the rostral medullary raphé contains sympathetic premotor neurons that control the thermoregulatory and heart outputs and is thought to be essential for expression of the hyperthermic and tachycardia responses to stressful stimuli [3, 24, 28–33]. Additionally, activation of 5-HT1A receptors in the medullary raphé has been shown to attenuate pharmacologically elicited increases in body temperature, including brown adipose tissue thermogenesis in anesthetized animals [34], as well as heart rate, including psychologically elicited tachycardia in conscious animals [35]. Due to these lines of evidence, it has been suggested that the medullary raphé serotonergic neurons are involved in the emotionally elicited autonomic physiological response.

In supporting this view, a recent study of ours found that intruder stress activates serotonergic neurons in the rostral medullary raphé [36]. It also shows that selective inhibition of these serotonergic neurons causes minor reduction in intruder-elicited tachycardia in mice, indicating that the medullary raphé serotonergic system somewhat contributes to stress-induced tachycardia. This could be via other 5-HT receptor subtypes or through the action of co-transmission from the serotonergic neurons since blockade of 5-HT2A receptors does not influence the tachycardia response to psychological stress. The spinal-projecting medullary raphé serotonergic neurons contain other transmitters [37].

The present study provides strong evidence that the serotonergic system is involved in stress-induced hyperthermia. The importance of the serotonergic system in thermoregulation is supported by a finding that inhibiting almost all serotonergic neurons in the brainstem decreases body temperature [38]. Interestingly, selective optogenetic inhibition of the medullary raphé serotonergic neurons does not cause a significant effect on intruder-elicited hyperthermia [36]. Since optical illumination cannot cover entire populations of serotonergic neurons in the medullary raphé, serotonergic neurons outside the optical irradiation region may be important for mediating the hyperthermia. Serotonergic neurons in the medullary raphé may have subpopulations depending on their sympathetic outflows in the same way as cardiovascular neurons found in the rostral ventrolateral medulla [39, 40]. Along with this, further investigation is required into other subpopulations of neurons found in different regions of the medullary raphé that influence thermoregulation during psychological stress. It is also possible that serotonergic groups of neurons found outside of this brain region are involved in the psychological-elicited hyperthermic response.

In summary, this study indicates that psychological stress causes activation of 5-HT2A receptors in the neural pathways that control thermogenesis. Further research is warranted to investigate specific brain regions expressing 5-HT2A receptors in the pathway.
